# Large gastric intramural hematoma mimicking a visceral artery aneurysm: a case report

**DOI:** 10.1186/s13256-018-1595-1

**Published:** 2018-03-07

**Authors:** Yuki Yoshioka, Kazuo Yoshioka, Shizuo Ikeyama

**Affiliations:** 1 Department of Emergency & Critical Care Medicine, Tokushima Red Cross Hospital, 103, Irinokuchi, Komatsushima-Cho, Komatsushima City, Tokushima 773-8205 Japan; 2 Department of Surgery, Taoka Hospital, 4-2-2, Bandai-Cho, Tokushima City, Tokushima 770-0941 Japan; 3 Department of Interventional Radiology, Taoka Hospital, 4-2-2, Bandai-Cho, Tokushima City, Tokushima 770-0941 Japan

**Keywords:** Gastric intramural hematoma, Gastric hematoma, Intra-abdominal aneurysm

## Abstract

**Background:**

Gastric hematoma is a rare disorder. Here we report a case of a large gastric intramural hematoma mimicking an impending rupture of a visceral artery aneurysm.

**Case presentation:**

A 60-year-old Japanese woman complained of left flank pain. Computed tomography with intravenously administered contrast agent showed a solid mass of 5 × 5 × 8 centimeter in the left middle abdominal quadrant. On completion of computed tomography, the working diagnosis was an impending rupture of an aneurysm located in a branch of the superior mesenteric artery. Transcatheter arterial embolization was performed, but angiography of the superior mesenteric artery and the inferior mesenteric artery did not indicate extravasation of the contrast agent and we did not observe any aneurysmal structure. We decided to perform surgery. The operational findings revealed that the mass was a gastric intramural hematoma.

**Conclusion:**

On encountering an intra-abdominal mass found to be attached to a gastric wall, a gastric intramural hematoma should be considered.

## Background

Gastric hematoma is a rare disorder. The precise incidence of gastric hematoma is unknown. Gastrointestinal intramural hematoma is well described in the esophagus and duodenum. The etiology of gastric intramural hematoma includes coagulopathy, anticoagulant therapy, trauma, and aneurysm. It can also be idiopathic or iatrogenic (endoscopic therapy and surgery) in nature. Here we report a case of a large gastric intramural hematoma mimicking an impending rupture of a visceral artery aneurysm.

## Case presentation

**Fig. 1:**
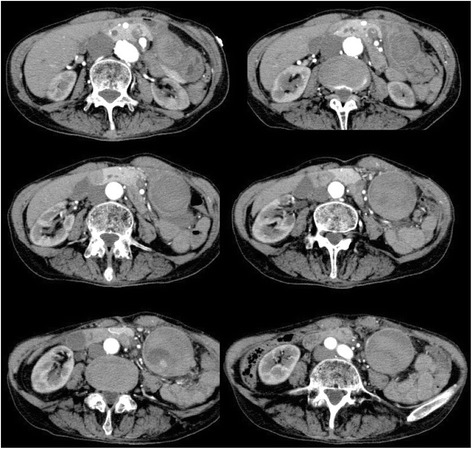
Contrast-enhanced computed tomography showed a large mass of 5 × 5 × 8 cm in the left middle abdominal quadrant. The mass was found to be attached to the gastric wall

**Fig. 2:**
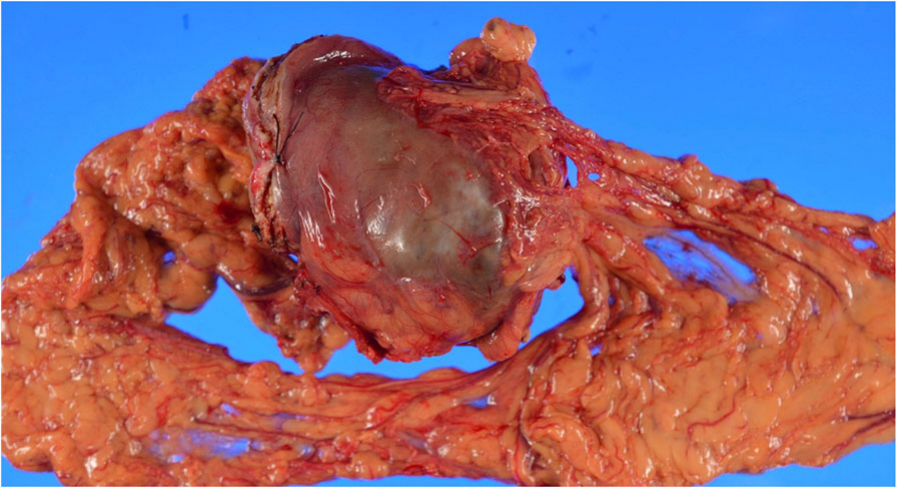
Gross appearance of the resected gastric hematoma. Mass was filled with clots

A 60-year-old Japanese woman was brought to the emergency department of our hospital, complaining of left flank pain. The pain had started the previous day and had gradually increased in severity. On examination, she was found to be alert, with a blood pressure of 173/101 mmHg, heart rate of 110 beats/minute, oxygen saturation of 95% (on room air), and respiratory rate of 20/minute. Her past medical history included myelodysplastic syndrome (MDS), hypertension, and cerebral infarction, leading to left hemiplegia. She had no history of pancreatitis and abdominal surgery. She was taking antihypertensive and acid-reducing medications, but no antiplatelet or anticoagulant medication. Her abdomen was flat, but there was moderate tenderness in her left flank. Her laboratory findings included hemoglobin level of 7 g/dL, hematocrit level of 20.8%, platelet count of 118,000/microL, prothrombin time of 13.3 seconds, activated prothrombin time of 31.9 seconds, and fibrinogen level of 209.4 mg/dL. Her normal hemoglobin level was approximately 8 g/dL. Blood chemistry findings were almost within normal limits. A computed tomography (CT) scan of her abdomen with intravenously administered contrast agent showed a solid mass of 5 × 5 × 8 cm in the left middle abdominal quadrant (Fig. 1). The mass was found to be fed by a branch of the superior mesenteric artery (SMA). The contrast agent leaked in the mass. An orally administered contrast agent was not used because she had nausea. On completion of CT, the working diagnosis was an impending rupture of an aneurysm located in a branch of SMA. We decided to perform a transcatheter arterial embolization (TAE); however, angiography of SMA and inferior mesenteric artery (IMA) did not indicate extravasation of the contrast agent. Her blood pressure remained stable, but her pulse rate was still fast. We decided to perform surgery in line with the diagnosis of an impending rupture of the visceral artery aneurysm. Her peritoneal cavity was entered with a midline incision, and there were no ascites. The mass was attached to the antrum of her stomach. There was a smooth surfaced mass at the greater curvature of the antrum. We suspected intra-tumor bleeding of the gastric submucosal tumor. We performed a wedge resection of her stomach including the mass with a 1 cm margin. The surgical specimen was opened and it revealed a large amount of clots (Fig. 2). There were no tumoral lesions.

Subsequently, pathological findings indicated that the mass had not included tumor cells and was simply filled with a large amount of clots. The etiology of the hematoma was not proven by pathological findings. She was admitted to the general surgical ward after surgery, and she was transfused with six units of packed red blood cells postoperatively. She was discharged from our hospital to a nursing home on day 63 after rehabilitation.

## Discussion

Here we report a case of large gastric intramural hematoma mimicking an aneurysm. Gastric hematoma is an uncommon disorder. This case was diagnosed only after surgical exploration, but it is possible that TAE could also be effective. At first, we did not recognize that the mass was such a large gastric intramural hematoma. The mass was found to be located far from the stomach. CT showed leakage of the contrast agent in the mass; therefore, we considered it to be a large partially thrombosed aneurysm. With CT angiography, the mass seemed to be fed by a branch of SMA. But angiography of SMA and IMA did not show the feeding artery or the leakage of contrast agents and any aneurysmal structure. An important finding of this study was that an angiography of the celiac artery would have provided a clearer diagnosis. Imaizumi *et al*. reported a case of gastric intramural hematoma successfully treated by TAE [1]. The mass size in that case was 17 × 8 cm. TAE was successfully performed in a branch of the left gastric artery.

Diagnosis of the gastric intramural hematoma was difficult. In this case, the preoperative diagnosis was an impending rupture of a visceral artery aneurysm. Even after surgery, the diagnosis was intra-tumor bleeding of the submucosal gastric tumor. Interpretation of the CT findings was difficult because of the location of the mass, which was found to be next to the greater curvature of the stomach, but located much inferior to the stomach. If the mass had been located in the upper quadrant, the diagnosis would have been easier. In the published case reports, there were similar difficulties in diagnosing gastric intramural hematomas. Out of the 10 cases in which surgeries were performed for gastric hematoma, the pre-surgical diagnosis was correct in four cases. In the other six cases, the pre-surgical diagnoses were gastric tumor in two [2, 3], pancreatic cyst in two [4, 5], and unknown in two cases [6, 7].

The etiology of the gastric intramural hematoma in this case is unknown. The pathological findings did not reveal the cause. We therefore considered this case to be an idiopathic hematoma. The causes of gastric hematoma include anticoagulant medication, coagulopathy, upper gastrointestinal endoscopy, trauma, and aneurysm; however, none of these apply to this patient. The medical history of this patient included MDS, which could have led to coagulopathy, but her platelet count of 118,000/microL was not low enough.

The chief complaints in the published case reports on gastric hematoma are various. Epigastralgia was the most frequent complaint. Abdominal pain and hematemesis were also common. In one case, in which the patient complained of chest pain, acute coronary syndrome was suspected and coronary angiography was performed. Subsequently, gastric intramural hematoma was diagnosed [8]. It should therefore be taken into consideration that an intragastric disease may cause chest pain.

Treatment of gastric hematoma was various, including an operation and TAE, endoscopic and percutaneous drainage, and conservative. All cases with coagulopathy and anti-coagulant therapy or anti-platelet therapy were successfully treated conservatively. The type of the surgery was total gastrectomy in three cases [7, 9, 10], partial gastrectomy in three cases [5, 11, 12], unknown in three cases [2–4], and exploratory laparotomy in one case [6].

## Conclusions

Here we reported a case of a large gastric intramural hematoma mimicking an aneurysm of a branch of SMA. On encountering an intra-abdominal mass found to be attached to a gastric wall, a gastric intramural hematoma is one of the differential diagnoses.
